# Supplementary material for: Combined transcriptome and proteome profiling reveals specific molecular brain signatures for sex, maturation and circalunar clock phase
Source: eLife. 2019 Feb 15;8:e41556. doi: 10.7554/eLife.41556 (PMC6377233; doi:10.7554/eLife.41556)
Supplement: Transparent reporting form [file elife-41556-transrepform.docx]

***eLife’s* transparent reporting form**

We encourage authors to provide detailed information *within their submission* to facilitate the interpretation and replication of experiments. Authors can upload supporting documentation to indicate the use of appropriate reporting guidelines for health-related research (see [EQUATOR Network](http://www.equator-network.org/%20)), life science research (see the [BioSharing Information Resource](https://biosharing.org/)), or the [ARRIVE guidelines](http://www.plosbiology.org/article/info:doi/10.1371/journal.pbio.1000412) for reporting work involving animal research. Where applicable, authors should refer to any relevant reporting standards documents in this form.

If you have any questions, please consult our Journal Policies and/or contact us: [editorial@elifesciences.org](mailto:editorial@elifesciences.org).

**Sample-size estimation**

- You should state whether an appropriate sample size was computed when the study was being designed
- You should state the statistical method of sample size computation and any required assumptions
- If no explicit power analysis was used, you should describe how you decided what sample (replicate) size (number) to use

Please outline where this information can be found within the submission (e.g., sections or figure legends), or explain why this information doesn’t apply to your submission:

- for transcriptomics and proteomics we used an n=3 Biological Replicates (each consisting of heads from 6 individual animals); as usual for theses large scale studies we did not pre-estimate a sample size and perform a power analysis. The analysis packages DESeq, EdgeR, limma, and ROTS are designed to provide statistically robust analyses even with low sample size, by “borrowing” information from all genes/proteins present in the data pool.

- likewise we didn`t perform an a-priori power estimation for qRT-PCR as these analyses were primarily performed to validate the expression profiles of selected transcripts found by quantitative RNA-Seq.

Information on sample sizes can be found in the Methods section:

RNA-Seq:	-Animal staging and head sampling

-RNA and protein extraction

-RNA-Seq library preparation and sequencing

-Fig. 1 – figure supplement 2e

Proteomics:	-Animal staging and head sampling

-RNA and protein extraction

-Mass spectrometry

-Fig. 1 – figure supplement 2f

qRT-PCR:	-Animal staging and head sampling

-RNA and protein extraction

-Statistics

**Replicates**

- You should report how often each experiment was performed
- You should include a definition of biological versus technical replication
- The data obtained should be provided and sufficient information should be provided to indicate the number of independent biological and/or technical replicates
- If you encountered any outliers, you should describe how these were handled
- Criteria for exclusion/inclusion of data should be clearly stated
- High-throughput sequence data should be uploaded before submission, with a private link for reviewers provided (these are available from both GEO and ArrayExpress)

Please outline where this information can be found within the submission (e.g., sections or figure legends), or explain why this information doesn’t apply to your submission:

Generally information on the above mentioned points can be found in the Methods section. For details, please refer to the points given above under “Sample size estimation”.

Information and accession numbers on transcriptome and proteome data are given under the point: Declarations 🡪 Availability of Data and Materials.

**Statistical reporting**

- Statistical analysis methods should be described and justified
- Raw data should be presented in figures whenever informative to do so (typically when N per group is less than 10)
- For each experiment, you should identify the statistical tests used, exact values of N, definitions of center, methods of multiple test correction, and dispersion and precision measures (e.g., mean, median, SD, SEM, confidence intervals; and, for the major substantive results, a measure of effect size (e.g., Pearson's r, Cohen's d)
- Report exact p-values wherever possible alongside the summary statistics and 95% confidence intervals. These should be reported for all key questions and not only when the p-value is less than 0.05.

Please outline where this information can be found within the submission (e.g., sections or figure legends), or explain why this information doesn’t apply to your submission:

Information on the above points can be found in the Methods section under the headings:

-Read mapping and differential expression analysis of RNA-Seq data and	selection of top regulated candidates for validation

-Differential Abundance Analysis of Proteomics data

-Data Analysis Methods RNA-Seq and proteomics functional analyses

-Statistics

as well as in the relevant figure legends.

(For large datasets, or papers with a very large number of statistical tests, you may upload a single table file with tests, Ns, etc., with reference to sections in the manuscript.)

**Group allocation**

- Indicate how samples were allocated into experimental groups (in the case of clinical studies, please specify allocation to treatment method); if randomization was used, please also state if restricted randomization was applied
- Indicate if masking was used during group allocation, data collection and/or data analysis

Please outline where this information can be found within the submission (e.g., sections or figure legends), or explain why this information doesn’t apply to your submission:

We performed transcriptomic and proteomic studies on different stages of maturation, sexes and lunar phases in a marine annelid. Within the stages, worms were distributed randomly to a given biological replicate. All analyses were done based on automated procedures (sequencing, mass spec, computer programs). The knowledge of the experimentors could thus not have interfered with or biased the results in any way. Randomisation and masking were therefore not undertaken.

**Additional data files (“source data”)**

- We encourage you to upload relevant additional data files, such as numerical data that are represented as a graph in a figure, or as a summary table
- Where provided, these should be in the most useful format, and they can be uploaded as “Source data” files linked to a main figure or table
- Include model definition files including the full list of parameters used
- Include code used for data analysis (e.g., R, MatLab)
- Avoid stating that data files are “available upon request”

Please indicate the figures or tables for which source data files have been provided:

Figures for which source data are provided:

Fig 1 🡪 Figure 1 source data 1 & 2

Fig 2 🡪 Figure 2 source data 1 - 4

Fig 3 including figure supplements

🡪 Figure 3 source data 1 - 14

Fig 4 including figure supplement

🡪 Figure 4 source data 1 **and** Figure 3 source data 2

Fig 5 including figure supplements

🡪 Figure 5 source data 1 - 21

Fig 6 🡪 Figure 6 source data 1 & 2 **and** Figure 3 source data 2

**and** Figure 5 source data 1

Fig 7 and figure supplement

🡪 Figure 7 source data 1 & 2 and Figure 3 source data 2

**and** Figure 5 source data 1
